# Chagas Disease and Transfusion Risk in Italy: The Results of a National Survey

**DOI:** 10.3390/pathogens11111229

**Published:** 2022-10-25

**Authors:** Ilaria Pati, Mario Cruciani, Francesca Masiello, Francesco Barone, Giacomo Silvioli, Massimo La Raja, Simonetta Pupella, Vincenzo De Angelis

**Affiliations:** National Blood Centre, Italian National Institute of Health, 00161 Rome, Italy

**Keywords:** Chagas disease, epidemiological surveillance, transfusion transmissible infection, *Tripanosoma cruzi*, blood transfusion safety

## Abstract

Background: Universal serological screening in endemic areas is essential for preventing Chagas disease transmission by transfusions, while in non-endemic areas, screening is provided only to donors exposed to the infection risk. In this respect, in order to ensure high and uniform standards of quality and safety of blood components, the Italian National Blood Centre conducted a survey to detect information on management of donors at risk of Chagas disease and on the current transfusion risk. Methods: The National Blood Centre conducted a survey on preventive measures for Chagas disease in the years 2020–2021. Results: Survey results are broadly representative of the national situation; out of 24,269 tested donors, only 15 donors were confirmed positive (0.4 out of 100,000 donors). This rate is lower than the number of positive donors (72/100,000) for transfusion transmissible infections (HIV, HBV, HCV, and *T. pallidum*) in the same period. Furthermore, the number of *T. cruzi* positive blood donors is lower than the *T. cruzi* positive subjects in the general population. Conclusions: In Italy, *T. cruzi* infection transfusion risk may be considered still very low, and this is confirmed by the absence of documented transfusion transmission.

## 1. Introduction

Chagas disease, also known as American trypanosomiasis, is vector-borne disease caused by *Trypanosoma cruzi,* a parasitic protozoan transmitted through the bite of hematophagous bugs (triatomines), found in various rural and suburban areas of Latin America. Transmission generally occurs due to the deposition, during the meal, of contaminated stool; *T. cruzi* is therefore able to enter the host through skin lesions, but also through the mucous membranes and ocular conjunctiva [1,2]. Infection can also be transmitted through transfusions, transplantation of organs, cells or tissues from an infected donor, by vertical (maternal–fetal) transmission, laboratory accidents or, more rarely, through the consumption of food and drinks prepared with fruits and vegetables contaminated by feces from sylvatic triatomines. There is no evidence of sexual transmission in humans; however, that is not excluded for other animal species [2,3].

Transfusion transmission of *T. cruzi* is well-documented and closely related to the type of blood component transfused, the immune status and the presence of antibodies in the recipient, and to donor parasitemia. *T. cruzi* can survive standard storage conditions for blood components (18 days at 4 °C for red blood cells, 250 days at 22 °C for platelets) and resist freezing (less than 24 h in frozen plasma). Based on these variables, it is estimated that 12–48% of subjects transfused with *T. cruzi*-positive blood components will develop the Chagas disease [3].

*T. cruzi* infection is curable if the person undergoes early treatment; the treatment can prevent or halt the progression of the disease, as well as mother-to-child transmission [1]. Currently there is no vaccine for Chagas disease; however, the actions taken in the past 20 years (vector control and serological screening of blood donors) to prevent the transmission of *T. cruzi* in endemic countries reduced the incidence and prevalence of the disease and contributed to disrupting transmission in some areas. The residual risk of transfusion transmission in these areas has been estimated around 1:200,000 transfusions [3,4].

Acute infections typically occur 1 week after exposure and can last up to 60 days. The incubation period ranges from 7 to 15 days for vector transmission; for post-transfusion infections, the incubation period is between 20 and 40 days, with a time interval of 8–120 days. Blood smears both stained and fresh can reveal the presence of trypomastigotes during the acute phase. At this stage, polymerase chain reaction (PCR) can be used for diagnosis. Acute infection is followed by a latent period, which can progress to chronic disease. The chronic phase begins when the parasitemia drops to undetectable levels, usually 4–8 weeks after infection; immunosuppression can reactivate the infection, causing high levels of parasitemia. During the chronic phase, parasitemia is usually undetectable; therefore, serological testing is considered the best strategy for blood-donor screening [1,3,4,5,6].

Universal serological screening in endemic areas is essential for preventing transfusion and organ-transplant transmission, detecting positive subjects, and guaranteeing access to treatment. In this regard, the U.S. Food and Drug Administration approved two tests for Chagas disease (the first in 2006, the second in 2010) validated for blood donors with the aim of reducing the risk of transfusion transmission [5,7,8].

For transfusion safety, it is certainly essential to conduct a medical history of the donor for detecting risk factors. In non-endemic areas, screening is provided only to donors exposed to the risk of infection: subjects born in (or with a mother born) or resident in countries where Chagas disease is endemic; and subjects transferred to endemic countries, or who have traveled or stayed in areas at risk (rural). The individuals considered at risk, according to the current Italian transfusion legislation and according to the European Directorate for the Quality of Medicines (EDQM) recommendations, can donate only with a negative validated test for the detection of anti-*T. cruzi* antibodies. Individuals with previous infection are permanently excluded from donating. These restrictions do not apply to donations of plasma for fractionation [6,7,9,10].

In order to increase sensitivity of diagnostic strategy, WHO indications recommend the use of two different serological tests; however, for blood donors, performing a single test is acceptable by main regulatory authorities in North America and Europe. In the event of discrepancies or inconclusive results, WHO recommends the use of a confirmatory testing, even in the absence of a consensus on the reference method as no test is considered the gold standard. PCR is not considered efficient in diagnosing chronic Chagas disease [6,7].

In order to gather information on present practices of transfusion-transmitted Chagas disease prevention and to ensure high and uniform standards of quality and safety of blood components, in view of the increasing relevance of the issue for the blood-transfusion system, the Italian National Blood Centre conducted a survey with the aim of detecting information on the management of donors at risk for Chagas disease and the current transfusion risk, considering the two-year period 2020–2021. Here, we report on the main findings of this survey.

## 2. Results

As reported in Table 1, the survey results are broadly representative of the national situation (81% of Blood Establishments (BEs) that perform blood and blood component collection/validation in Italy), with an average regional participation equal to 89% of involved BEs.

The subsequent data processing was carried out considering the 219 BE respondents out of the 272 who perform collection/validation of blood and blood components.

A total of 61% of BEs perform the screening test of the donor at risk for Chagas disease using an immunoenzymatic assay at the time of donation; 37% of the BEs apply for a temporary deferral and perform the test before donation only at a later stage; the remaining 2% used both approaches or did not provide any feedback.

In 2020, 15,261 donors, corresponding to 0.8% of the total Italian blood donors in the same year, were deemed at risk, and therefore tested for anti-*T. cruzi* antibodies, while for the year 2021, the donors tested were 9,008, corresponding to 0.5% of the total Italian blood donors in 2021. With respect to the number of blood donors tested, 0.12% had a positive/inconclusive anti-*T. cruzi* antibody test both in 2020 (19/15,261 donors) and in 2021 (11/9008 blood donors). Of these, 53% (10/19 blood donors) in 2020 and 45% (5/11 blood donors) in 2021 were confirmed positive. Of note, although we do not have sensitivity and specificity data of the diagnostic screening test and confirmatory test, we can assume that 47% and 55% of the screening tests performed in 2020 and 2021 were false-positive (Table 2).

Some donors were deferred; those at risk for Chagas disease (travel or birth in risk areas) who did not have a screening test for anti-*T. cruzi* antibodies numbered 6 (0.33/100,000 blood donors) in 2020 and 14 (0.75/100,000 blood donors) in 2021 (Table 2).

As reported in Table 3, most of the detected positive blood donors (9 out of 15) were born in endemic areas; the remaining 6 positive donors reported travel in endemic areas as a risk factor.

Given the small number of confirmed positive donors or deferred donors compared to the general blood donor population, we can assume that the lack of data, on average, for about 39% of Lombardy, Marche, Campania, Sardinia and Sicily region BEs, has no impact on overall survey results.

Table 4 shows the data detail for each region and for each year considered. Out of a total of 24,269 tested donors, only 15 donors belonging to the regions of Abruzzo (1 donor), Campania (1 donor), Emilia Romagna (1 donor), Latium (6 donors), Lombardy (4 donors), and Tuscany (2 donors) were confirmed positive for the entire two-year period. On average, about 0.3 out of 100,000 donors per region are positive for each year.

Twenty donors were deferred without testing in the Sardinia and Sicily regions (Table 4).

**Figure 1:**
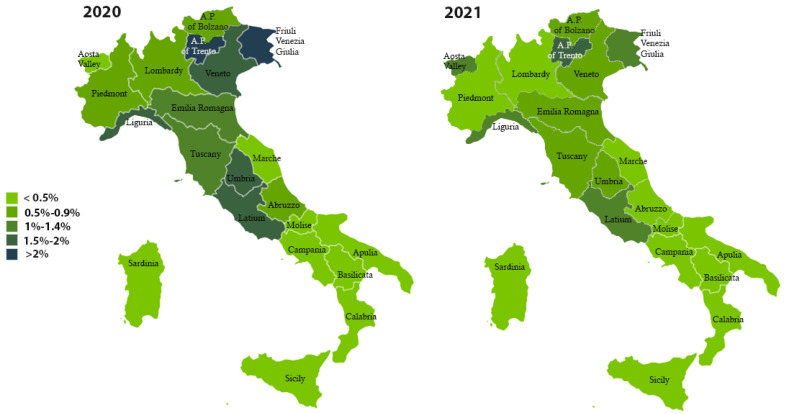
Percentage distribution of blood donors tested for anti-*T. cruzi* antibodies, years 2020–2021.

As shown in Figure 1, the A.P. of Trento and Friuli Venezia Giulia tested the largest number of donors in 2020 (2.7% and 2.3%, respectively). Additionally in 2021, the A.P. of Trento reported the highest number of donors tested (2%) compared to other Italian regions.

**Figure 2:**
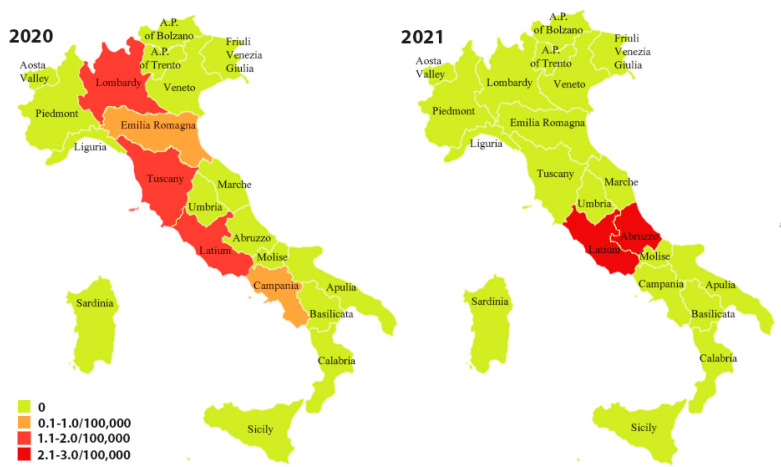
Distribution (on 100,000 donors) of confirmed anti-*T. cruzi* antibody positive blood donors, years 2020–2021.

In 2020, the regions with the highest number of positivity were Lazio, Lombardy, and Tuscany (1.4/100,000 blood donors). The Lazio Region had the highest number of positivity in 2021 (2.8/100,000 blood donors) (Figure 2).

## 3. Discussion

It is estimated that 6–7 million people worldwide are infected with *T. cruzi*; the highest number of infections is recorded in Latin America, where the disease is endemic, followed by the United States, with over 325,000 estimated cases, and Europe [1,11]. Due to migratory flows, currently most of the infected people live in urban contexts and, increasingly, move to non-endemic countries such as the United States, Canada, some African countries and many European countries. Spain is the European non-endemic country with the highest number of cases (estimated 70,000 to 76,000 cases), followed by Italy (estimated 9,000 to 10,000 cases), France, and Germany (from 2000 to 3000 estimated cases). In these areas, in the absence of the vector, the disease is transmitted mainly transplacentally, through transfusion or transplantation of organs from infected donors [1,6,12]. Infected subjects are usually asymptomatic in the chronic phase (70–80% of cases); therefore, if they are candidates for blood donation, they can represent a risk for transfusion safety [3,13]. In this case, two different serological tests with simultaneous detection of anti-*T. cruzi* antibodies are indicated for the infection diagnosis and total blood-donor screening. The enzyme-linked immunosorbent test (ELISA) and immunofluorescent antibody test (IFA) are commonly used [14].

The survey, conducted by the Italian National Blood Centre, provided important information on the epidemiology of Chagas disease in the blood-donor population and also about the current transfusion risk. The donors tested for Chagas disease in the years 2020–2021, were, respectively, 0.8% and 0.5% of the total donors. Of these, only 0.06% in 2020 (66/100,000 donors tested) and 0.05% in 2021 (55/100,000 donors tested) were confirmed positive. Although the SARS-CoV-2 outbreak may have affected travel and therefore the number of subjects at risk, the data obtained from a survey in 2018 show that the ratio between tested and confirmed positive donors is even lower (0.03% of tested) compared to the results of the current survey. Overall, in the two-year period 2020–2021, the donors confirmed positive for the anti-*T. cruzi* antibodies were 0.06% of the donors considered at risk. Compared to the total number of donors, the donors considered at risk for Chagas disease were 655 out of 100,000, while the confirmed positives were 0.4 out of 100,000. Of note, when the prevalence of a disease is very low, as in the case of *T. cruzi* infection in Italy, the positive predictive value of a test will also be low, particularly for a screening test with low specificity. Actually, there was a high rate of false-positive results with the screening test not validated by the confirmatory test; as reported in Table 2, about 50% of the initial positivity has not been confirmed. More than 50% of positive subjects reported birth in endemic areas (9/15 positive blood donors confirmed). As an average result among the Italian regions, less than 1% of total donors were tested for Chagas disease and only 2.6 donors/1,000,000 could be confirmed positive.

According to the results of the Chagas survey, confirmed positive donors in 2020–2021 represented 0.0004% of the total donors (0.4/100,000), compared with the percentage of blood donors resulting positive for transfusion-transmissible infections (TTIs, HIV, HBV, HCV, and *T. pallidum*) detected in the same period (72/100,000, respectively 4/100,000 for HIV, 30/100,000 for HBV, 10/100,000 for HCV, and 28/100,000 for *T. pallidum*). Furthermore, the number of *T. cruzi*-positive blood donors was also lower than the estimate of positive subjects in the general population: out of a national average of 9200 cases in the general population, the positive subjects were about 15 out of 100,000 people. As the donor population is a selected population, this could explain the different number for positivity compared to the general population. The survey results suggest that the number of blood donors considered at risk for Chagas disease was overestimated compared to the real incidence of positivity. The testing is preferable to permanent blood-donor deferral, which could include suitable subjects; however, it should be reserved for donor candidates who actually have a history of risk factors for Chagas disease. Considering that Italy is not an endemic country, the stringent criteria applied for blood-donor selection justify the survey results and support the lack of cases of transfusion transmission. As a result, in Italy *T. cruzi* infection transfusion risk may be considered still very low.

## 4. Materials and Methods

The National Blood Centre, with the support of the Regional Blood Coordination Centres, administered the survey “National survey on preventive measures for Chagas disease in the transfusion field” to all the Italian BEs that collect and validate blood and blood components. The survey covered the two-year period 2020–2021 and concerned the detection of Chagas disease positivity in blood donors at risk: donors born in (or with a mother born) or reside in countries where Chagas disease is endemic, or transferred to endemic countries, or that traveled or stayed in areas at risk (rural) [10]. According to current transfusion regulations, donors at risk for Chagas disease can only donate with a negative validate *T. cruzi* antibody test [10]. Participating BEs provided the number of *T. cruzi* antibody-test positive donors, reported on their Information Systems. Since all donors with inconclusive/positive serological screening test were subjected to confirmatory tests, the BEs also provided the number of confirmed positive donors.

The data, collected through the surveys, were processed in an aggregate way to provide the national data. A descriptive statistical analysis was conducted using the Microsoft Excel program.

## Figures and Tables

**Table 1:** Percentage of participant Blood Establishments for each Italian Region/Autonomous Province.

Region/A.P.	Participating BEs (%)
Abruzzo	100%
Aosta Valley	100%
Apulia	100%
Basilicata	100%
Calabria	100%
Campania	47%
Emilia Romagna	100%
Friuli Venezia Giulia	100%
Latium	100%
Liguria	100%
Lombardy	54%
Marche	58%
Molise	100%
A.P. of Bolzano	100%
A.P. of Trento	100%
Piedmont	100%
Sardinia	60%
Sicily	55%
Tuscany	93%
Umbria	100%
Veneto	100%
Mean (SD)	88.9% (±19.2%)

A.P.: Autonomous Province; BEs: Blood Establishments.

**Table 2:** Description of main survey results, years 2020–2021.

	2020	2021
Total donors (N.)	1,845,142	1,863,050
Tested donors for Chagas disease (N.)	15,261	9008
Tested/total donors (%)	0.8%	0.5%
Positive-inconclusive donor test (N.)	19	11
Positive-inconclusive/tested donors (%)	0.12%	0.12%
Positive donors confirmed (N.)	10	5
Positive confirmed/positive-inconclusive donor test (%)	53%	45%
Donors excluded without testing (N.)	6	14

N.: number.

**Table 3:** Risk factors for Chagas disease detected in positive blood donors confirmed.

	Birth in Endemic Areas (N.)	Travel History in Endemic Areas (N.)
2020	5	5
2021	4	1
Total	9	6

**Table 4:** Description of main survey results for *T. cruzi* per region, years 2020–2021.

	Blood Donors—2020			Blood Donors—2021		
Region/A.P.	Tested (% Total Donors)	Confirmed (per 100.000)	Excluded (per 100.000)	Tested (% Total Donors)	Confirmed (per 100.000)	Excluded (per 100.000)
Abruzzo	207 (0.5%)	0	0	177 (0.4%)	1 (2.5)	0
Aosta Valley	68 (2.0%)	0	0	57 (1.5%)	0	0
Apulia	157 (0.1%)	0	0	127 (0.1%)	0	0
Basilicata	33 (0.2%)	0	0	20 (0.1%)	0	0
Calabria	0	0	0	0	0	0
Campania	5 (0.01%)	1 (0.7)	0	12 (0.01%)	0	0
Emilia Romagna	2169 (1.3%)	1 (0.6)	0	903 (0.5%)	0	0
Friuli Venezia Giulia	1120 (2.3%)	0	0	550 (1.2%)	0	0
Latium	2300 (1.6%)	2 (1.4)	0	1356 (1.0%)	4 (2.8)	0
Liguria	834 (1.7%)	0	0	615 (1.3%)	0	0
Lombardy	1803 (0.6%)	4 (1.4)	0	901 (0.3%)	0	0
Marche	155 (0.3%)	0	0	138 (0.3%)	0	0
Molise	0	0	0	0	0	0
A.P. of Bolzano	108 (0.6%)	0	0	102 (0.6%)	0	0
A.P. of Trento	542 (2.7%)	0	0	419 (2.0%)	0	0
Piedmont	758 (0.6%)	0	0	497 (0.4%)	0	0
Sardinia	15 (0.03%)	0	0	74 (0.1%)	0	6 (10.6)
Sicily	12 (0.01%)	0	6 (3.6)	4 (0.002%)	0	8 (4.9)
Tuscany	1721 (1.2%)	2 (1.4)	0	1182 (0.9%)	0	0
Umbria	406 (1.5%)	0	0	199 (0.7%)	0	0
Veneto	2848 (1.6%)	0	0	1675 (0.9%)	0	0
Total	15,261	10	6	9008	5	14
Mean (SD)	0.9% (±0.8%)	0.3 (±0.5)	0.2 (±0.7)	0.6% (±0.6%)	0.3 (±0.8)	0.7 (±2.4)

A.P.: Autonomous Province. For each region/A.P. and for each year, it is reported: the number of blood donors tested, with the percentage of the total blood donor population (second and fourth columns); the number of confirmed positive donors, with the value per 100,000 blood donors (third and fifth columns); the number of donors excluded, with the value per 100,000 blood donors (fourth and sixth columns).

## Data Availability

The data supporting the reported results can be found at National Blood Centre, Italian National Institute of Health, Rome, Italy.
